# Th17-cells in depression: Implication in multiple sclerosis

**DOI:** 10.3389/fimmu.2022.1010304

**Published:** 2022-09-14

**Authors:** Mikhail Melnikov, Anna Lopatina

**Affiliations:** ^1^ Department of Neuroimmunology, Federal Center of Brain Research and Neurotechnology of the Federal Medical-Biological Agency of Russia, Moscow, Russia; ^2^ Department of Neurology, Neurosurgery and Medical Genetics, Pirogov Russian National Research Medical University, Moscow, Russia; ^3^ Laboratory of Clinical Immunology, National Research Center Institute of Immunology of the Federal Medical-Biological Agency of Russia, Moscow, Russia

**Keywords:** Th17-cells, neuroinflammation, depression, antidepressants, multiple sclerosis

## Abstract

Depression is one of the most common neuropsychological symptoms of multiple sclerosis. However, in addition to mood disorder, depression can also influence on multiple sclerosis course. The mechanism of this dependence is not fully understood. The recent studies suggest the possible common immune mechanisms in the pathogenesis of depression and multiple sclerosis. In particular, it was shown that along with biogenic amines disturbance, neuroinflammation also play an important role in the pathogenesis of depression. Significant attention is drawn to Th17-cells subsets, which are considered as critical players in the pathogenesis of inflammatory diseases of the central nervous system, including multiple sclerosis. This brief report reviews the literature data on the role of neuroinflammation in the reciprocal influence of multiple sclerosis and depression with focus on Th17-cells, which may underlie pathogenetic mechanisms of both this diseases.

## Introduction

Multiple sclerosis (MS) is a demyelinating, neurodegenerative and autoimmune disease of the central nervous system (CNS) which affects presumably young people. Despite the fact that etiology of MS is still unclear it is well known that the psycho-emotional stress can influence on MS pathogenesis and course (1, 2). Depression is one of the most common neuropsychological symptoms of MS. The prevalence of depression in MS patients could reach from 30 to 70% (3, 4). However, depression is not only the cause of mood disorder in MS. It was shown that depression could promote exacerbations of the disease and, therefore, aggravate its course (5). The influence of depression on MS pathogenesis is also confirmed by the impact of controlled stress therapy on MS course (treatment with antidepressants, lifestyle modification, usage of coping strategies, etc.) (6–8).

At the same time, the mechanism of this dependence is not fully understood. On the one hand, the impact of depression on MS course could be mediated by its influence on the adherence to long-term pathogenetic treatment of MS. It was shown that MS patients with mood or anxiety disorders are almost five times more likely to have reduced adherence to the disease-modifying therapy (DMT), compared with MS patients without neuropsychological impairments (9, 10). In addition, depression can induce the development of other neuropsychological disorders, such as cognitive impairment, fatigue, which can also decrease the adherence to pathogenetic therapy of MS (11, 12).

On the other hand, the common immune mechanisms underlying depression and MS pathogenesis could explain this. The recent evidence suggest that neuroinflammation, along with an imbalance of biogenic amines, may also play an important role in the pathogenesis of depression (13). Particular attention is drawn to Th17-cells subsets, which are considered as critical players in the pathogenesis of inflammatory diseases of the CNS, such as MS, neuromyelitis optica spectrum disorder, Parkinson’s disease and other (14). It was shown that Th17-cells also participate in the pathogenesis of depression (15).

**Figure 1:**
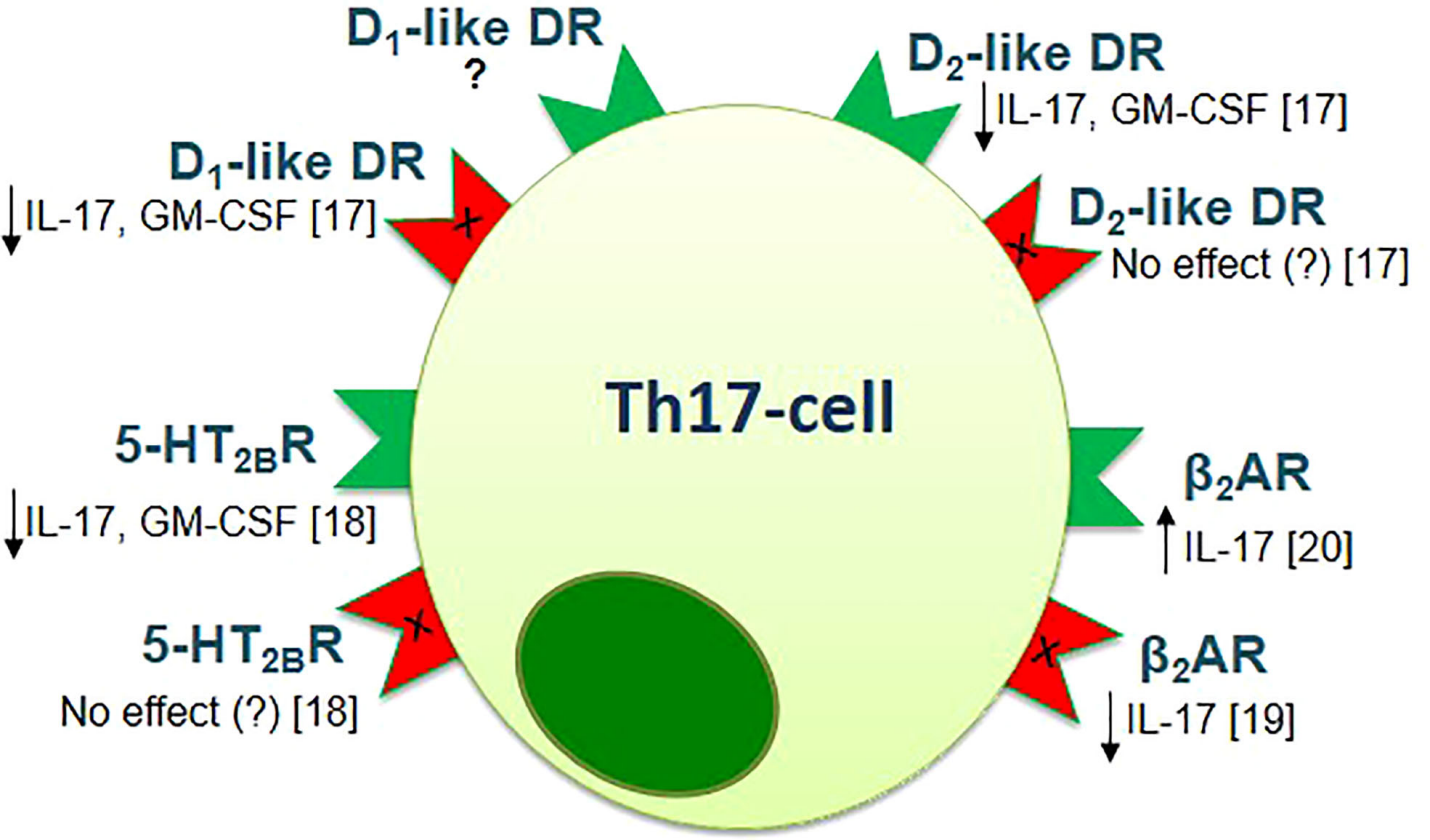
The effect of activation (green) or blockade (red) of biogenic amine receptors on the function of human Th17-cells [adapted from Melnikov et al., 2021 (20)]. Data obtained from the anti-CD3/CD28-activated purified CD4+ T-cells of patients with relapsing-remitting MS and healthy subjects [Melnikov et al., 2022 (17); Sviridova et al., 2021 (18); Melnikov, Rogovskii et al., 2022 (19)] or from the anti-CD3/CD28-activated PBMCs or purified CD3+CD4+CCR6+ Th17-cells of healthy subjects [Carvajal Gonczi et al., 2017 (21)]. D1- or D2-like DR, D1- or D2-like dopaminergic receptor; 5-HT2BR, 5-hydroxytryptamine receptor 2B; β2AR, β2-adrenergic receptor.

It is important to note that biogenic amines, which are involved in the pathophysiology of the neuropsychological disorders, also have a modulating effect on the cells of both innate and adaptive immune systems, including subsets of T-helper cells and can affect the MS pathogenesis by modulating the Th17-immune response (16–21). In particular, the inhibitory effect of dopamine, norepinephrine, and 5-HT was shown on Th17-cytokines production by activated CD4^+^ T-cells in patients with relapsing-remitting MS and in healthy subjects (17–19). It is important to note, that these effects were not associated with inhibition of cell viability or proliferative response, which suggests the clear modulatory effect of biogenic amines on Th17-cells (17–19). The types of receptors, which could mediate this effect, were identified (17–19). In addition, the direct effect of activation or blockade of biogenic amine receptor on Th17-cells function was shown (**Figure 1**).

It can be assumed that Th17-cells may play a central role in the mutual relationship between depression and MS, while targeting the biogenic amine receptors allows modulating the Th17-dependent neuroinflammation in depression and MS.

This brief report reviews the literature data on the role of Th17-cells in the pathogenesis of depression. The influence of depression on Th17-immune response in multiple sclerosis is also discussed.

## The role of neuroinflammation in the pathogenesis of depression: Focus on Th17-cells function

Despite, the fact that depression is one of the most common of the psychiatric diseases, the pathogenetic mechanism of depression is still discusses. Currently, there are some basic theories of the pathogenesis of depression: classical «biogenic amines theory», «cytokine theory», and «kynurenine theory» (22, 23). It is most likely that these hypotheses are interdependent. In particular, it was shown that almost all immune cells are express receptors for dopamine, norepinephrine, epinephrine, and serotonin (5-hydroxytryptamine [5-HT]), suggesting the immunomodulatory effect of biogenic amines (24). Furthermore, the ability of peripheral blood mononuclear cells (PBMCs) and CD4^+^ T-cells subsets to produce catecholamines was also reported. On the other hand, the cytokines, such as interferon-γ (IFN-γ) and IFN-β can regulate this production (25). The involvement of neuroimmune interactions in the pathogenesis of depression is also confirmed by clinical studies. Thus, the prevalence of depression is significantly higher in patients with chronic inflammatory diseases, including autoimmune diseases (26). Herewith, the treatment of these patients with cytokines may increase the depression severity (27). In this regard, much attention is drawn up to the role of immune system in the development of depression as well the influence of biogenic amines on the functioning of immune cells (16, 28).

Th17-cells are one of the subpopulations of CD4^+^ T-helper cells, with pro-inflammatory phenotype. Th17-cells produce such cytokines as interleukin-17 (IL-17), IL-21, IL-22, granulocyte- and granulocyte-macrophage colony-stimulating factors (G-CSF and GM-CSF). The Th17-cells are known to be involved in the autoimmunity (29). However, in the previous years, Th17-cells attracted attention due to their role in the development of inflammatory diseases of the CNS (14). It was shown that Th17-cells can increase the permeability of the blood-brain barrier due to production of IL-17 and migrate into the CNS due to expression of the chemokine receptor 6 (CCR6) (30, 31).

The recent evidence suggests that Th17-cells play an important role in the pathogenesis of depression. Thus, Hong et al. (2013) reported that in chronic unpredictable mild depression caused by stress, behavioral changes in mice might correlate with the imbalance between Th17- and Treg-cell subsets (32). Beurel et al. (2013), showed that the number of Th17-cells (CD4^+^IL-17^+^ T-cells) was higher in the brains of mice with learned helplessness compared to the non-depressed mice, while the number of Treg-cells (CD4^+^FoxP3^+^ T-cells) was comparable between the groups. The numbers of Th17-cells was also higher in the spleens of learned helpless mice than in control group. In correspondence with these data, transfer of Th17-cells to wild-type mice was sufficient to promote depression-like behaviors, including learned helplessness. Conversely, RORγT-knockout mice demonstrated resistance to learned helplessness, which suggests the role of Th17-cells in promotion of this behavior. The same results were obtained using another model of depression (mice were subjected to restraint stress for two weeks): chronic restraint stress resulted in increased brain levels of Th17-cells (33). In subsequent study the authors (2018) showed that Th17-, but not Th1-cells promote depressive-like behavior in wild-type mice, confirming their previously results. In addition, they found increasing expression of CCR6 in CD4^+^IL-17A^+^ T-cells in the hippocampus of learned-helpless mice (34).

In a recent study, Kim et al. (2021) found the increasing levels of IL-17 in the brains and blood serum in mice with depression-like behavior (cumulative mild prenatal stress). They also found the increased level of RORγt as well as CD4^+^IL-17^+^ T-cells in the brains of mice with cumulative mild prenatal stress compared to the control group. At the same time, after treatment with anti-IL-17 antibody, depression-like behavior was evaluated in mice with cumulative mild prenatal stress. The IL-17 level in the brains of these mice was also significantly reduced (35).

The enhancing of Th17-cells functioning was observed in mice with autoimmune diseases and depression. Thus, Nadeem et al. (2017) showed that the depression-like state caused by psoriasis in mice is accompanied by increased systemic/neuronal expression of IL-17A, which was associated with increased NFκB/p38MAPK signaling and inflammatory mediators in brain regions, and depression-like symptoms. Accordingly, administration of IL-17A had effects similar to psoriasis-like inflammation on neurobehavioral and NFκB/p38MAPK pathways, while blockade of both NFκB and p38MAPK reduced IL-17A levels associated with depression-like behavior. At the same time, anti-IL-17A-antibody reduced depression-like symptoms, as well as NFκB/p38MAPK-signaling (36). In line with these data, Griffiths et al. (2017) reported that the treatment with a high-affinity monoclonal antibody to IL-17A ixekizumab reduced depressive symptoms in patients with psoriasis. Herewith, the authors did not find any influence of etanercept (tumor necrosis factor inhibitor) treatment on depressive symptoms, which suggests the pathogenetic role of IL-17A in the development of depression in patients with psoriasis (37). However, according to another study, etanercept can improve fatigue and symptoms of depression in patients with psoriasis (38).

The increasing of Th17-immune response in mice with depression-like behavior corresponds to the data of patients with major depressive disorder (MDD). Thus, in the study by Chen et al. (2011) it was shown that percent of Th17-cells in the culture of PBMCs is higher in patients with MDD than in healthy subject, while the percent of Treg-cells, on the contrary, is lower. These data correspond to the level of RORγt (transcription factor of Th17-cell) mRNA expression in peripheral blood lymphocytes and serum level of IL-17 (39). The same results were obtained by Davami et al. (2016), who showed increased serum level of IL-17 in MDD patients compared with healthy subjects (40). At the same time in another study, the increased serum levels of IL-17 in MDD patients were not confirmed. In addition, the IL-17 level did not change during treatment with antidepressants (41).

Bliźniewska-Kowalska et al. (2020) showed increase in the level of Th17-cytokines in peripheral blood in patients with depression (42). In the subsequent study (Gałecka et al, 2021), the authors confirmed their previously results. They showed increase in serum IL-17 and IL-23 levels as well as IL-17 and IL-23 mRNA expression in peripheral blood lymphocytes of patients with MDD (n=190) compared with the control group (n=100) (43). In correspondence with these data, Alvarez-Mon et al. (2021) found increase in serum IL-17 level in patients with MDD compared to healthy subjects. They also found that the percentage of the total IL-17 producing CD4^+^ T-cells among PBMCs was also higher in MDD patients (44).

Jha et al. (2017) reported that higher baseline plasma level of IL-17 were selectively associated with greater symptomatic reduction in depressed patients treated with a combination of bupropion (atypical antidepressant)-escitalopram (selective serotonin reuptake inhibitors [SSRI]), which suggests a prognostic factor for determination of peripheral IL-17 for medication selection (45).

In whole, the *in vivo* and *in vitro* studies allow to consider Th17-cells as new players in the pathogenesis of depression (46). Most probably, their role in the development of mood disorders can increase in cases of comorbidity with autoimmune diseases (47).

## Th17-immune response in MS patients with depression

The relationship between Th17-immune response and depression in MS has not been sufficiently studied. However, some studies reported the increasing of Th17-cells function in patients with relapsing-remitting MS and depression. In particular, in one of our studies we found that the percentage of circulating CD4^+^CD26^+^CD161^+^CD196^+^ Th17-cells and production of IL-17 and IFN-γ by anti-CD3/anti-CD28-stimulated PBMCs was higher in patients with relapsing-remitting MS and depression (n=20) compared to patients without depression (n=25) or to the control group (healthy subjects, n=20). However, it should be noted, that some MS patients with depression were examined during relapse of the disease. We also found that 5-HT at a concentration of 10^–4^ M reduces IL-17 and IFN-γ by activated PBMCs without affecting cell proliferation or cell viability in all groups. We did not find any difference in the inhibitory effect of 5-HT on cytokine production between the groups (48).

The study by Sales et al. (2021) showed an increase in the levels of IL-6 and IL-1ß in blood plasma in patients with relapsing-remitting MS (n=25) and MDD compared with MS patients without MDD (n=25). They also found that production of IL‐1β, IL‐6, IL-17, IL-22, IL-23, and GM‐CSF by activated PBMCs was also higher in MDD MS patients. At the same time, there were no differences in the production of IFN-γ and IL-12 by activated PBMCs between the groups, which suggests the activation of Th17-branch of immune system in MDD MS patients (49). Thus, IL-17, IL-22, and GM-CSF are produced by Th17-cells, while IL-6, IL-1β, and, especially, IL-23 are required for Th17-cells differentiation (29). In contrast, the production of anti-inflammatory cytokine IL-10 was lower in MDD MS patients (48). The percentage of IL‐17‐producing CD4^+^ and CD8^+^ T-cells positive for TLR2 was also significantly higher in the cultures from MDD MS patients, which corresponds to increasing IL-17 production by activated PBMCs in MDD MS patients. The same results were obtained when the authors studied cytokine production by activated purified CD4^+^ and CD8^+^ T-cells (49).

The authors also investigated the effect of 5-HT (200 ng/ml) on cytokine production by activated PBMCs and CD4^+^ and CD8^+^ T-cells in MS patients with and without MDD. It was found that 5‐HT reduced IL‐1β, IL‐6, IL‐23, and IL‐17 production by activated PBMCs in both groups and GM‐CSF in MDD MS patients that corresponds to our results. 5-HT also suppressed IL-6 and IL-17 production by activated CD4^+^ and CD8^+^ T-cells in both groups. Finally, Sales and coauthors showed the influence of depression treatment with SSRI fluoxetine (20 mg/day) on Th17-cells function in MDD MS patients. They found that after six month of therapy with fluoxetine the percentage of circulating CD4^+^ and CD8^+^ T-cells decreased. Furthermore, fluoxetine decreased the production of IL‐1β, IL‐6, and IL‐17 by activated CD4^+^ and CD8^+^ T-cells, suggesting an anti-inflammatory effect of treatment with fluoxetine on Th17-immune response in relapsing-remitting MS patients with MDD (49).

A subsequent study by Sacramento et al. (2022) confirmed the effect of MDD on Th17-cells function in MS. Again, they found an increase in plasma levels of IL-1β, IL-6, and IL-17 in MDD MS patients (n=20) compared to MS patients without depression (n=20). In correspondence with previously data, the production of IL-6, IL-21, IL-22, GM-CSF, and IFN-γ by anti-CD3/anti-CD28-activated PBMCs was higher in MDD MS patients, while the production of IL-10, conversely, was lower. In response to anti-CD3/anti-CD28 beads, the proportion of CD4^+^IL-17^+^ and CD8^+^IL-17^+^ T-cells was also higher in the MDD MS patients group. At the same time, the percentages of IL-10-producing and FoxP3-expressing CD4^+^ T-cells (with CD3/CD28-stimulation) was higher in MS patients without depression (50).

Notably, that enhancing of Th17-immune response is observed in MS patients and EAE mice with other neuropsychological symptoms, such as fatigue and cognitive impairments, which also are associated with depression (51, 52). In particular, Alvarenga-Filho et al. (2017) found that production of Th17-cytokines IL-17, IL-22, and GM-CSF by anti-CD3/CD28-activated PBMCs is higher in relapsing-remitting MS patients with fatigue (n=15) compared to MS patients without fatigue (n=15) (51). Another study showed a dose-dependent effect of IL-17 on hippocampal long-term potentiation through the activation of IL-17A receptor and p38MAPK in mice with EAE, while the lack of IL-17A ameliorates EAE-related cognitive deficits (52). Kant et al. (2018) reported that Th17-cells might infiltrate brain regions implicated in the pathophysiology of obsessive-compulsive disorders and could be responsible for the compulsive behavior in mice with EAE (53).

In summary, despite the fact that the number of studies on the impact of depression on Th17-cells in MS is currently relatively small, the existing data suggests that depression may enhance Th17-immune response in MS. Considering the crucial role of Th17-cells in the pathogenesis of MS, the enhancing of Th17-immune response in depression may underlie the mechanism of the influence of depression on MS course.

## Discussion

The possible immune mechanisms of the relationship between depression and MS are still being discussed (54, 55). In addition to Th1-cells and microglial cells, the analyzed data suggest a central role of Th17-cells in the development of neuroinflammation in MS and depression and may explain their reciprocal influence. The involvement of Th17-cells in the pathogenesis of depression and MS could be mediated by several mechanisms. In particular, Th17-cells produce pro-inflammatory cytokines and may increase the blood-brain barrier permeability promoting their (and other immune cells) penetration into the CNS, where Th17-cells could activate microglial cells, which may present antigens of the CNS leading to further recruitment of Th17-cells (13, 56, 57). In addition, the microglial cells activated by IL-17 may produce pro-inflammatory cytokines such as IL-1β, IL-6, and IL-23, which are necessary for Th17-differentiation (13, 56, 57).

In this regard, Th17-cells could be considered as a dual therapeutic target allowing the both MS and depression courses to be improved. Furthermore, the involvement of Th17-cells in the pathogenesis of depression allows proposing that clinical efficacy of antidepressants, at least partially, could be mediated by their anti-inflammatory effect on Th17-cells function suggesting the possible influence of antidepressants on MS pathogenesis and course. Thus, it was shown that treatment with antidepressants such as SSRIs, selective serotonin norepinephrine reuptake inhibitors (SSNRIs), and tricyclic antidepressants (TCA) can prevent the EAE development and reduce the EAE severity (6). Moreover, the anti-inflammatory effect of SSRIs was also shown in MS, psoriasis and rheumatoid arthritis (58, 59). On the other hand, the influence of treatment with anti-IL-17-antibodyes on depression in patients with various autoimmune diseases was shown (37, 47, 60). An anti-depressive effect was observed for DMT of MS glatiramer acetate (61), which also suppresses Th17-cells and dendritic cells mediated Th17-immune response (62, 63). In addition, according to some authors, the inhibitors of p38MAPK pathway, which are involved in Th17-cell activation, also could be considered as potential therapeutics for the treatment of depression (64, 65).

It is important to note, that antidepressants can affect inflammation directly in the CNS, while the ability of monoclonal antibodies to migrate through blood-brain barrier is limited, which suggests their effect primarily on the periphery (66). In this regard, the potential ability of antidepressants to modulate Th17-mediated neuroinflammation in MS has drawn a great attention. In some studies, the suppressive effect of various classes of antidepressants on Th17-cells function was shown (**Table 1**). However, the impact of antidepressants on Th17-immune response in EAE and MS has not been sufficiently studied. In a recent study, Sviridova et al. (2021) showed the inhibitory effect of fluoxetine on IL-17, IFN-γ, and GM-CSF production by anti-CD3/CD28-activated CD4^+^ T-cells in patients with relapsing-remitting MS and healthy subjects (18), which confirms the anti-inflammatory effect of fluoxetine on Th17-cells in MS (49). 5-HT_2B_-receptor were also found to be involved in the modulating effect of fluoxetine on Th17-cells in MS patients (18).

**Table 1:** The influence of antidepressants on Th17-cells function.

Drug	Disease	The effect on Th17-cells	Authors
Combined treatment with desloratadine (histamine H1-receptor antagonist) and nortriptyline (tricyclic-antidepressant [TCA])	Mice with relapsing-remitting EAE	The combined *in vivo* treatment with deloratadine (3 mg/kg) and Nortriptyline (10 mg/kg) decreases the IL-17 production by PLP or anti-CD3-activated splenocytes and anti-CD3-activated lymph node cells in mice with EAE. Nortriptyline (0–1mM) dose-dependently reduced IL-17 production by PLP and OVA activated lymph node cells and anti-CD3/anti-CD28-activated naive CD4^+^ T-cells.	Podojil et al., 2011 (67)
Desipramine (TCA)	Mice with allergic rhinitis (AR) and depression	Desipramine administration (3 mg/kg, 10 mg/kg or 30 mg/kg) decreased CD4^+^IL-17^+^/CD4^+^ T-cells ratio in AR mice spleen cells.	Zhang et al., 2013 (68)
Amitriptyline (TCA)	Mice with *C. rodentium* infection	Treatment with amitriptyline (180 mg/l *via* drinking water for 14 days before infection and 10 days during infection) enhance frequencies of CD4^+^IL-17^+^ and CD4^+^IFN-γ^+^ T-cells cells through acid sphingomyelinase inhibition.	Meiners et al., 2019 (69)
Paroxetine (selective serotonin reuptake inhibitor [SSRI])	Rats with collagen-induced arthritis (CIA)	The *in vitro* treatment of murine CD3^+^ T-cells with paroxetine (10^–5^ M) prevents Th17-cells differentiation under IL-6/TGF-β/IL-23 induction *via* GRK2 inhibition. The *in vivo* treatment of CIA rats with paroxetine (15 mg/kg/day) significantly reduced the splenic Th17-cells.	Hu et al., 2021 (70)
Fluoxetine (SSRI)	RRMS with depression	The treatment with fluoxetine (20 mg/day) during 6 months reduced the IL-17 production by Pam3C or LPS-activated CD4^+^ and CD8^+^ T-cells.	Sales et al., 2021 (49)
Fluoxetine	RRMS	Fluoxetine (10^–6^ M) suppresses IL-17, IFN-γ, and GM-CSF production by anti-CD3/CD28-activated CD4^+^ T-cells in RRMS patients and healthy subjects. 5-HT_2B_-receptor antagonist reduced the inhibitory effect of fluoxetine on cytokine production in MS patients.	Sviridova et al., 2021 (18)

Considering the anti-inflammatory effect of antidepressants and their safety and tolerability profiles, the repurposing of serotonergic, dopaminergic, and noradrenergic drugs for pathogenetic treatment of MS is discussed (6, 71–74). It is probably, that antidepressants could be tested as an additional treatment to the first-line DMT of MS (71–74). In particular, according to our recent retrospective pilot study, the addition of therapy with fluoxetine in patients with relapsing-remitting MS with a suboptimal response to the first-line DMT (IFN-β or glatiramer acetate) can reduce the disease activity, preventing the switching to the more aggressive second-line DMT (75). Although this is preliminary data, which needs to be confirmed in prospective studies with a control group, the results of this analysis are in line with the data of other studies and suggest the potential clinical efficacy of antidepressants as a pathogenetic treatment of MS (20).

## Conclusion

Taken together, the recent evidence suggests an important role of Th17-cells in the pathogenesis of both MS and depression. Along with biogenic amines, Th17-cells could be considered as direct mediators in the neuroimmune interaction between these diseases, while modulation of Th17-cells function by targeting the biogenic amine receptors may be a promising additional therapeutic approach in the treatment of MS and depression.

## Author contributions

MM: conceptualization, methodology, investigation, writing – original draft preparation. AL: investigation, writing – original draft preparation. All authors agree to be accountable for the content of the work.

## Funding

The work was financially supported by the Russian Foundation for Basic Research (RFBR) and Moscow city Government according to the project № 21-315-70014. The funders had no role in study design, data collection and analysis, decision to publish, or preparation of the manuscript.

## Conflict of interest

The authors declare that the research was conducted in the absence of any commercial or financial relationships that could be construed as a potential conflict of interest.

## Publisher’s note

All claims expressed in this article are solely those of the authors and do not necessarily represent those of their affiliated organizations, or those of the publisher, the editors and the reviewers. Any product that may be evaluated in this article, or claim that may be made by its manufacturer, is not guaranteed or endorsed by the publisher.
